# Septic shock caused by *Elizabethkingia miricola* in an elderly trauma patient: a case report and systematic literature review

**DOI:** 10.3389/fmed.2025.1561379

**Published:** 2025-05-07

**Authors:** Xiaoxiao Mao, Xiao Feng, Chaoyang Li, Xiupeng Xu, Lin Zhao, Jia Xu, Liqing Bi, Zhen Yue

**Affiliations:** ^1^Department of Neurosurgery, The First Affiliated Hospital of Nanjing Medical University, Nanjing, China; ^2^Department of Critical Care Medicine, The First Affiliated Hospital of Nanjing Medical University, Nanjing, China; ^3^Department of Pathology, Jiangsu Province Hospital of Chinese Medicine, Nanjing, China

**Keywords:** *Elizabethkingia miricola*, pulmonary infection, septic shock, case report, systematic review

## Abstract

**Objective:**

*Elizabethkingia miricola* is a rarely encountered pathogen in clinical settings, predominantly causing infections in immunocompromised individuals. To advance the understanding of *E. miricola* infection, we present a case of *E. miricola* infection and conduct a literature review.

**Methods:**

We report a case of pulmonary infection caused by *E. miricola* in a 90-year-old trauma patient, marking the first documented instance of treatment with eravacycline. We also conducted a systematic review of the relevant literature. A comprehensive search was performed using the PubMed and Web of Science databases up to November 2024. A qualitative synthesis was conducted on all available case reports and case series related to *E. miricola* infections.

**Results:**

A total of 63 cases from 21 studies were included in this systematic review. According to these case reports, infections caused by *E. miricola* most commonly occur in the lungs (34/63), bloodstream (6/63), and urinary tract (4/63). Risk factors for *E. miricola* infection include immunodeficiency, prolonged hospitalization in the intensive care unit (ICU), prolonged mechanical ventilation, and the use of broad-spectrum antibiotics. Notably, a considerable proportion of cases (17/63) are hospital-acquired.

**Conclusion:**

*Elizabethkingia miricola* represents a rare but highly lethal opportunistic pathogen. Early identification and treatment with sensitive antibiotics are required to improve the prognosis of patients. The present case and literature review provide options for the diagnosis and treatment of similar cases in the future and serve as a reference for preventing and controlling the occurrence and spread of nosocomial infections.

**Systematic review registration:**

https://pubmed.ncbi.nlm.nih.gov/, https://www.webofscience.com/wos/.

## 1 Introduction

*Elizabethkingia miricola* is a species within the genus *Elizabethkingia*, characterized as Gram-negative, non-fermentative, non-motile, and non-spore-forming. It was initially isolated from condensation water on the Mir space station in 2003 (1). This bacterium is widely distributed in freshwater and soil and is frequently isolated from amphibians, particularly frogs. The first documented case of human infection was reported in 2008 in a patient with lymphoma who presented with recurrent fever following stem cell transplantation and chemotherapy, with *E. miricola* identified in both sputum and blood samples (2). In recent years, with the extensive use of broad-spectrum antibiotics and the increase in invasive medical procedures, the incidence of nosocomial infections attributed to *E. miricola* has been rising annually.

Since the genus *Elizabethkingia* usually carries multiple drug resistance genes, the bacteria in the genus often exhibit multidrug resistance, including *E. miricola*. This drug resistance poses great challenges in treatment and causes increased mortality in patients infected with *E. miricola*. Carrying two types of *β*-lactamase genes, *E. miricola* commonly exhibits resistance to the majority of *β*-lactam and carbapenem antibiotics, except piperacillin-tazobactam. As a *β*-lactamase inhibitor, tazobactam binds tightly to the β-lactamase produced by *E. miricola*, inhibiting the activity of the enzyme and allowing piperacillin to maintain its antibacterial activity. Previous case reports have demonstrated that *E. miricola* is susceptible to fluoroquinolones, sulfonamides (e.g., trimethoprim/sulfamethoxazole), and tetracyclines. In general, these antibiotics have often been used in the treatment of *E. miricola* infections (3–6).

## 2 Case presentation

### 2.1 Basic information

A 90-year-old man was admitted to the hospital after sustaining multiple traumatic injuries in a traffic accident that occurred 6 days prior. On 6 October 2024, the patient was transferred to a local hospital in a comatose state. An emergency computed tomography (CT) scan revealed contusions and lacerations in the cerebrum and multiple fractures of the C4 vertebra. Additionally, an X-ray examination identified a displaced fracture of the right lower tibia and fibula. An emergency surgical procedure was performed to debride and suture the right lower tibia and fibula. Postoperatively, the patient remained in a comatose state and manifested a persistent fever, which led to his admission to the neurosurgery intensive care unit at our hospital on 12 October 2024. Upon admission, the physical examination revealed a body temperature of 36.5°C. The patient was comatose, as indicated by a Glasgow Coma Scale (GCS) score of 2 + T + 2. Following clinical examination, the patient was tracheally intubated and mechanically ventilated. Auscultation detected scattered moist rales in both lungs.

### 2.2 Treatment history

On 13 October 2024, laboratory tests showed a white blood cell count of 8.55 × 10^9^/L (normal: 4–10 × 10^9^/L), hemoglobin of 73 g/L (normal: 130–175 g/L), platelets of 133 × 10^9^/L (normal: 125–350 × 10^9^/L), and creatinine of 45 μmol/L (normal: 44–132 μmol/L). After admission, the patient was administered mechanical ventilation and cefoperazone/sulbactam. On 17 October 2024, a sputum culture revealed the presence of carbapenem-resistant *Klebsiella pneumoniae*, susceptible to tigecycline and with intermediate susceptibility to colistin. Consequently, the treatment regimen was adjusted to include the intravenous administration of colistin sulfate in conjunction with the inhalation of colistin methanesulfonate. Until 19 October, the patient’s peak body temperature remained below 38.5°C. However, on 20 October, the patient presented an elevated peak temperature of 40.1°C, accompanied by a substantial decline in 24-h urine output to 1,100 mL, and creatinine clearance decreased to 51.7 mL/min (normal: 80–120 mL/min). Considering the inadequate response to anti-infective treatment, the therapeutic regimen was altered to intravenous administration of tigecycline. By 21 October, the patient remained in fever and septic shock, as indicated by a quick Sequential Organ Failure Assessment (qSOFA) score of 2. A drop in blood pressure necessitated aggressive fluid resuscitation and continuous intravenous infusion of norepinephrine to maintain hemodynamic stability. Renal insufficiency was further evidenced by a decrease in creatinine clearance to 35.5 mL/min. A bedside X-ray demonstrated consolidation in both lower lobes of the lungs, with greater severity observed in the right lung.

On 21 October, a bronchoscopy with alveolar lavage revealed purulent sputum in both lower lobes. Semi-quantitative cultures (using the quadrant streak method on a plate containing blood agar) obtained from the bronchoalveolar lavage fluid showed moderate growth of two distinct Gram-negative bacteria, namely *Elizabethkingia miricola* and *Pseudomonas aeruginosa*, identified via matrix-assisted laser desorption/ionization time-of-flight mass spectrometry (MALDI-TOF/MS). The results of the drug susceptibility test suggested that *Pseudomonas aeruginosa* was susceptible to cefoperazone/sulbactam, meropenem, and other antibiotics, while *E. miricola* exhibited resistance to multiple antibiotics, as shown in Table 1. The susceptibility results were interpreted according to the CLSI M100 (2024) breakpoints for non-*Enterobacteriaceae* bacteria (7). The presence of *E. miricola* was confirmed through metagenomic next-generation sequencing (mNGS) of bronchoalveolar lavage fluid, yielding 151,173 sequences of *E. miricola* and 11,367 sequences of *P. aeruginosa*.

**Table 1:** Antibiotic resistance of the tested *Elizabethkingia miricola.*

Antibiotic class	Tested antibiotics	Results	Breakpoints (ug/mL)	Interpretation
Penicillins/*β*-Lactamase inhibitor combinations	Ticarcillin/Clavulanate acid	≥128	≤16/2	R
	Piperacillin/tazobactam	≥128	≤16/2	R
Third-generation cephalosporins	Ceftazidime	≥64	≤8	R
Fourth-generation cephalosporins	Cefepime	≥32	≤8	R
Cephalosporin/β-Lactamase inhibitor combinations	Cefoperazone/sulbactam	32	≤16	I
Monobactams	Aztreonam	≥64	≤8	R
Carbapenems	Imipenem	≥16	≤4	R
	Meropenem	≥16	≤4	R
Aminoglycosides	Amikacin	≥64	≤16	R
	Tobramycin	≥16	≤4	R
Tetracyclines	Doxycycline	2	≤4	S
	Minocycline	≤1	≤4	S
	Tigecycline	4		I
Quinolones	Ciprofloxacin	≥4	≤1	R
	Levofloxacin	≥8	≤2	R
Sulfonamides	Trimethoprim/sulfamethoxazole	40	≤38/2	S
Polymyxins	Colistin	≥16		R

R: resistance; I: intermediate sensitivity; S: sensitivity.

On 22 October, the patient’s procalcitonin level was measured at 1.84 ng/mL (normal: 0–0.05 ng/mL), and a complete blood count revealed a white blood cell count of 18.0 × 10^9/L with a neutrophil percentage of 80.6% (normal: 40–75%). The patient presented with a peak body temperature of 39.2°C and was administered intravenous eravacycline at a dosage of 50 mg every 12 h in conjunction with intravenous cefoperazone/sulbactam at 3 g every 12 h. Subsequently, a gradual decrease was observed in the patient’s peak temperature, white blood cell count, and inflammatory markers. On 28 October, a sputum culture showed no growth of *E. miricola*. The patient remained in a coma and suffered from anemia, along with a decline in platelet count beginning on 26 October. A platelet transfusion was administered, and human immunoglobulin was provided to block the antibodies. On 3 November, a routine blood test revealed a platelet count of 3 × 10^9^/L. The patient’s family decided to discontinue treatment and discharged the patient from the hospital. A telephone follow-up later confirmed the patient’s death.

## 3 Materials and methods

### 3.1 Study design and objectives

We conducted a comprehensive search, selection, data extraction, and bias assessment of published literature on human infections caused by *E. miricola*. The objective of this systematic review was to assess and deepen the understanding of the clinical characteristics associated with this rare microorganism, thereby facilitating more effective treatment strategies in clinical practice.

### 3.2 Search strategy

A comprehensive literature search was performed using the PubMed and Web of Science databases, covering the period from their inception until 30 November 2024. The search term was “*Elizabethkingia miricola,*” with no language restrictions applied. The inclusion criteria for this systematic review are articles reporting at least one case of *E. miricola* infection, with eligibility confined to studies based on human cases.

### 3.3 Data extraction

The literature search and selection were independently performed by two researchers, Xiaoxiao Mao and Zhen Yue, who also extracted the relevant data from the studies. We evaluated basic information about the studies (first author, publication year) and patient characteristics (age, gender, site of infection, clinical manifestations, underlying diseases, antimicrobial susceptibility results, anti-infective treatment procedures, and prognosis). Any disagreements between the researchers were resolved through consensus. This study was conducted in accordance with the 2020 Preferred Reporting Items for Systematic Reviews and Meta-Analyses (PRISMA) guidelines (8). Given the study design, ethical committee approval was not required. Informed consent for the case report was obtained from the patient’s family.

### 3.4 Bias assessment

The risk of bias in the case reports and case series incorporated into the study was systematically evaluated utilizing the Joanna Briggs Institute (JBI) Critical Appraisal Checklist for Case Reports and Case Series (9). The items used to evaluate each study are as follows: demographic characteristics of the patients, patient medical history, current clinical condition of the patients, diagnostic tests or assessment methods and their results, interventions or treatment procedures, clinical condition after intervention, adverse events (harms) or unexpected events, and lessons learned. According to the recommendations of the JBI Critical Appraisal Checklist, for each evaluation criterion of the case report, “Yes” indicates a low risk of bias, while each occurrence of “No” affects the overall quality of the case report. If “Unclear” appears, it indicates an unknown risk of bias. The bias assessment was conducted independently by two researchers (Xiaoxiao Mao and Zhen Yue), with disagreements resolved through consensus.

## 4 Results

### 4.1 Study selection

**Figure 1:**
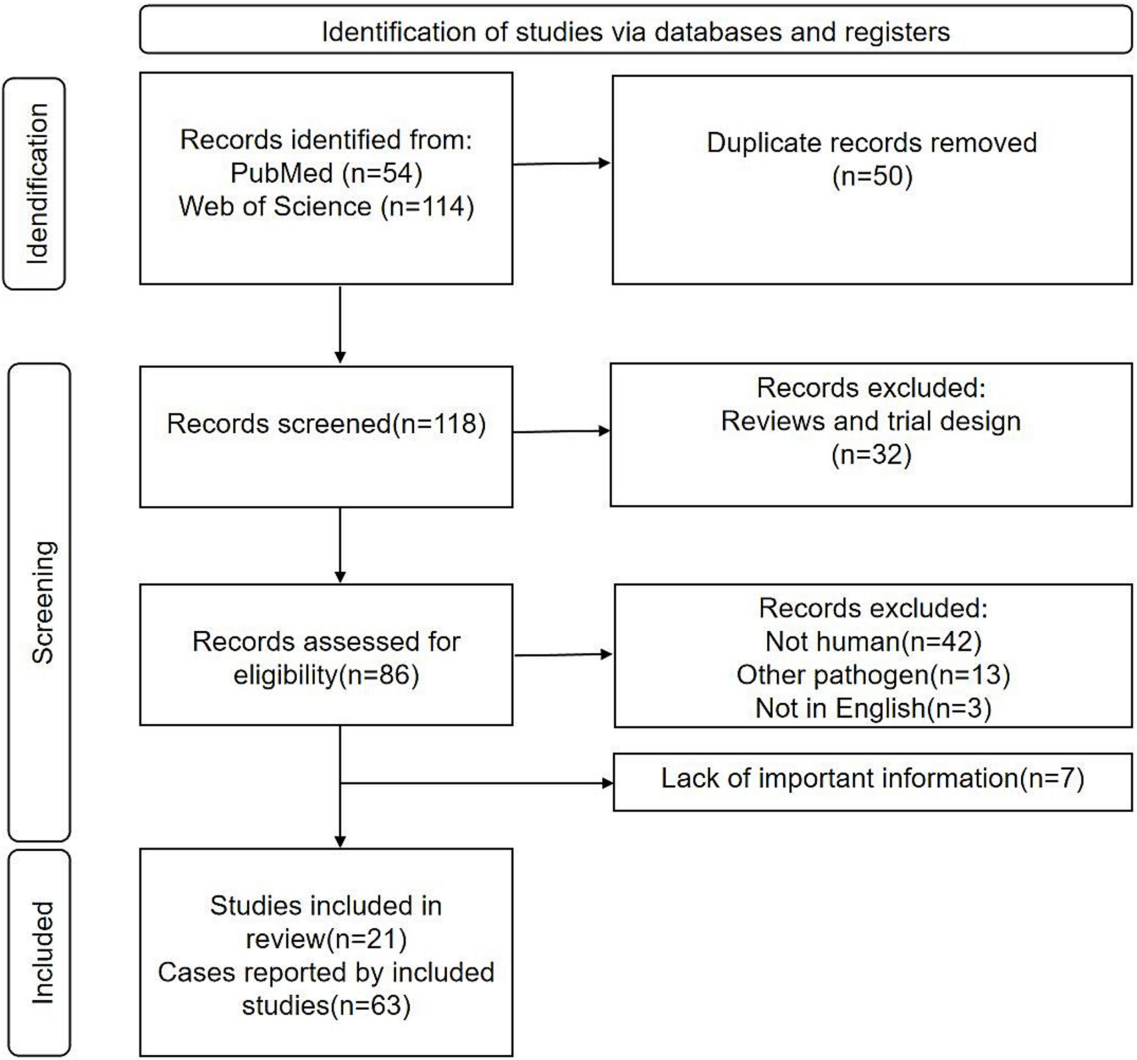
PRISMA flow diagram of article inclusion.

As shown in Figure 1, the PRISMA flowchart delineates the selection process for the studies included in this review. Utilizing the specified keywords, we initially identified 54 articles from the PubMed database and 114 articles from the Web of Science database. After removing duplicates, we retained a total of 118 unique articles. We subsequently excluded 33 articles comprising basic research, review articles, and conference abstracts; 42 articles on non-human infections; 13 articles on other pathogens; and 3 articles not published in English. Subsequently, we carefully reviewed the remaining 27 articles, of which we excluded 7 articles due to the absence of critical information. Ultimately, 21 studies were included in the final analysis, encompassing a total of 63 cases of *E. miricola* infection.

### 4.2 Study characteristics

All included studies were published between 2018 and 2024 (Supplementary Table 1), with 18 studies being single-case reports and 3 studies being case series. After the first human *E. miricola* infection was reported in 2008, the second case was not reported until 2015, with 95% (60/63) reported after 2017.

These case reports were distributed across different countries, predominantly in temperate regions (57/63). Two distant and unrelated case series were recorded in Spain, and one occurred in South Korea, with the majority of cases being pulmonary infections (38/45). Bloodstream infections and urinary tract infections were also prevalent, while both intracranial *E. miricola* cases occurred in China. In Spain, an outbreak in the ICU occurred within 9 months (10). In another case series in Spain (11), the diagnostic period was concentrated in 15 months. The case series in South Korea spanned from 2011 to 2014.

### 4.3 Characteristics of patients with *E. miricola* infection

The age distribution of individuals infected with *E. miricola* was found to be extensive, ranging from a 2-year-old child to 82-year-old adults, suggesting that the pathogen poses a risk across diverse age demographics. A few infected individuals (6/63) were under 40 years of age, indicating higher susceptibility among the elderly. The majority of the infected population was found to consist of male patients.

Infected individuals often had pre-existing medical conditions, such as chronic renal insufficiency, pancreatitis, and immunodeficiency caused by various factors. In the ICU outbreak reported by Soler-Iborte et al. (10), the primary disease in the majority of patients was COVID-19 (8/10), and all patients were treated with steroids and mechanical ventilation.

*Elizabethkingia miricola* infections present with non-specific symptoms such as fever, inflammation, elevated white blood cell count, and lung exudates. The bacteria often lead to persistent lung colonization, recurrent pneumonia, and sepsis, with limited treatment options due to antibiotic resistance. This results in a poor prognosis and high mortality, especially in pulmonary cases.

### 4.4 Sites of infection

*Elizabethkingia miricola* is capable of infecting multiple organ systems, with pulmonary infections being the most prevalent, accounting for 54.0% (34/63), followed by bloodstream infections at 9.5% (6/63) and urinary tract infections at 6.3% (4/63). Studies on antibiotic susceptibility suggest that fluoroquinolones (such as ciprofloxacin and levofloxacin) exhibit favorable antimicrobial efficacy against *E. miricola*. Moreover, piperacillin-tazobactam, aminoglycoside drugs (such as gentamicin and amikacin), sulfonamide drugs (such as co-trimoxazole), and tetracycline drugs (such as tigecycline and minocycline) are also regarded as effective therapeutic options for infections caused by this bacterium. The overall prognosis for patients infected with *E. miricola* was found to be poor, with a general mortality rate of approximately 17.5% (11/63). A higher mortality rate was observed among patients with pulmonary infections, reaching 29.4% (10/34).

### 4.5 Sources of *E. miricola* infection

A considerable proportion of cases were observed to be nosocomial infections (17/63). In the majority of cases, *E. miricola* was not the initial pathogen responsible for the development of tracheobronchitis, pneumonia, and other infections. The interval between the patient’s hospitalization and the diagnosis of *E. miricola* infection was usually quite long. In one reported case series, the average time interval was 26.4 days (10).

According to the case reports with clear results of drug susceptibility tests, *E. miricola* is most sensitive to quinolones (31 cases), followed by piperacillin-tazobactam (26 cases), tetracyclines (11 cases), and trimethoprim-sulfamethoxazole (9 cases). Correspondingly, the most prevalent anti-infection regimens used in these cases are trimethoprim-sulfamethoxazole (12 cases), quinolones (10 cases), piperacillin-tazobactam (8 cases), and tetracyclines (4 cases).

### 4.6 Quality appraisal

The overall quality of the included studies was deemed satisfactory; therefore, the majority of the articles were determined to have a low risk of bias. The quality assessment for individual case reports is detailed in Supplementary Table 2, while the evaluation for case series is presented in Supplementary Table 3.

## 5 Discussion

In recent years, there has been an upward trend in infections attributed to *E. miricola*, particularly among immunocompromised individuals. Historically, the majority of infections have been observed in patients who were hospitalized for extended periods of time, especially in intensive care units. Patients with pulmonary *E. miricola* infections generally have a prolonged history of mechanical ventilation. Our review indicates that our patient represents the first documented case of a pulmonary infection with *E. miricola* following multiple traumatic injuries and is also the oldest reported patient. The patient was immunocompromised due to trauma-induced stress, was in a comatose state, required mechanical ventilation, and was unable to expectorate spontaneously, which resulted in pulmonary infection. Although *E. miricola* can be a colonizing bacterium, in this case, it caused obvious symptoms of septic shock and pulmonary infiltration, substantiated by bronchoalveolar lavage fluid culture and mNGS. Therefore, it is not considered a case of colonization.

In this case, both *E. miricola* and *P. aeruginosa* precipitated severe pulmonary infection and septic shock. The high sequence count of *E. miricola*, combined with its ability to induce septic shock, signifies its role as the primary pathogen. The improvement in clinical symptoms and laboratory markers, in conjunction with the eradication of *E. miricola*, indicated that eravacycline exerted a beneficial effect on the infection. Nevertheless, it should be noted that concomitant treatment of *P. aeruginosa* and other confounding factors may have influenced the overall outcome.

As previously noted, a significant proportion of *E. miricola* cases were found to be nosocomial infections. *E. miricola* is a widespread pathogen found in soil and water. Within a hospital setting, the organism can be transmitted among patients, with nosocomial infections being attributed to various factors. These include contamination of medical equipment and transmission via the hands of healthcare workers. Patients are often immunocompromised due to underlying conditions and treatments such as antimicrobial therapy, corticosteroid therapy, or prolonged mechanical ventilation, which diminishes their airway clearance capacity, increasing their susceptibility to infection. Soler-Iborte et al. reported a case series of a nosocomial *E. miricola* outbreak in the ICU, noting that infections persisted despite standard precautions and contact precautions (10). Thorough disinfection eventually halted new cases, suggesting that *E. miricola* may be transmitted through the hospital environment in the hospital. Similarly, the case in the current report was a nosocomial infection. After the identification of *E. miricola*, routine nosocomial infection monitoring was carried out in the hospital wards. However, during the monitoring of the ward environment for nosocomial infections and in the pathogen culture of surrounding patients, *E. miricola* was not detected, thereby leaving the origin of the infection uncertain. Our patient had been on mechanical ventilation for 15 days before 21 October, and the ventilator is considered to be the most likely source of infection. Our findings suggest that multifaceted interventions are critical to reducing *E. miricola* transmission in hospitals. These interventions should encompass medical surface disinfection, proper management of high-risk infection sources (e.g., water tanks), and strict adherence to hand hygiene and contact precautions. Moreover, high-risk populations, particularly tracheally intubated patients, require intensified airway management protocols. Therapeutic regimens should be tailored through comprehensive patient evaluation, emphasizing antimicrobial stewardship to minimize broad-spectrum antibiotic overuse while optimizing nutritional interventions. Further investigations are warranted to improve *E. miricola* infection control in the future.

The presence of *E. miricola* was confirmed through sputum culture and identified using MALDI-TOF/MS and mNGS. Based on drug susceptibility testing, the tetracycline antibiotic eravacycline was selected for the anti-infective treatment. Following targeted treatment, *E. miricola* was successfully eradicated from the patient’s sputum specimens. Despite the patient ultimately being discharged against medical advice due to other severe complications, the anti-infective therapy proved effective. The patient, an elderly individual, experienced a consciousness disorder following multiple traumas, required long-term mechanical ventilation, and developed a pulmonary infection caused by carbapenem-resistant *Enterobacteriaceae* (CRE), for which broad-spectrum antibiotic treatment was administered. These factors collectively constitute risk factors for *E. miricola* infection. Onyia Opota et al. reported the case of an 82-year-old patient who, following cervical spinal surgery, experienced paraplegia and recurrent pulmonary infections (4). Upon admission to the ICU, cultures of the patient’s airway secretions revealed the presence of *Stenotrophomonas maltophilia* and *E. miricola*. Despite the administration of antimicrobial therapy, the patient succumbed rapidly. In conclusion, pulmonary infections caused by *E. miricola* pose a significant threat to elderly patients.

*Elizabethkingia miricola* demonstrates multidrug resistance due to the presence of multiple drug resistance genes. It harbors two types of *β*-lactamase genes, conferring resistance to the majority of β-lactam and carbapenem antibiotics. *E. miricola* exhibits low susceptibility to aminoglycosides and is intrinsically resistant to polymyxins. Additionally, it is susceptible to fluoroquinolones, including levofloxacin and moxifloxacin, in addition to minocycline. Susceptibility to tigecycline is difficult to predict, according to inconsistent reports (3). In this instance, *E. miricola* exhibited resistance to *β*-lactam and fluoroquinolone antibiotics while demonstrating susceptibility to tetracycline antibiotics.

According to CLSI-M100 2024, the MIC susceptibility and resistance breakpoints for doxycycline and minocycline are 4 mg/L and 16 mg/L, respectively. Tigecycline breakpoints for non-*Enterobacteriaceae* bacteria are not available. In this case, the MIC of the *E. miricola* strain to the second-generation tetracyclines was 2 mg/L, suggesting that this strain may not carry the genes responsible for resistance to tetracyclines. Given the structural and functional similarities within the tetracycline family, it is reasonable to infer that it may also be susceptibility to eravacycline. Consequently, eravacycline was administered as an anti-infective treatment. Following the onset of sepsis, the patient developed bone marrow suppression, manifesting as anemia and thrombocytopenia, along with persistent consciousness disorder and respiratory failure, which did not significantly improve with treatment. As a result, the patient’s family opted to discontinue further medical interventions. A study published in 2024 reported a young woman with a pulmonary infection from *Elizabethkingia anophelis* after heart valve surgery, which improved after eravacycline treatment (12). This case report suggests that eravacycline demonstrated good efficacy in the treatment of *E. anophelis* infections. Eravacycline, a synthetic fluorocycline, is approved for treating infections caused by multidrug-resistant bacteria. It achieves high concentrations in lung tissue, and its successful application in treating infections caused by other drug-resistant Gram-negative bacteria supports its potential utility in the treatment of *Elizabethkingia* infections. Prospective studies are needed to assess its efficacy and safety in treating *E. miricola* infections.

This case underscores the importance of early, targeted anti-infective therapy to achieve a favorable prognosis for patients. A critical initial step involves the accurate identification of *Elizabethkingia miricola*. As reported by Han M-S. et al., the MALDI-TOF/MS system demonstrated high accuracy in identifying *Elizabethkingia meningitis*, *Elizabethkingia mansonia*, and *Elizabethkingia miricola*, but significant errors were observed in identifying *Elizabethkingia miricola* with the Bruker Biotyper microbial identification system and the Vitek 2 GN card system. Notably, mNGS is increasingly pivotal in microbial identification due to its superior accuracy and efficiency (5). Additionally, mNGS is capable of detecting drug resistance genes associated with *E. miricola*, thereby providing a critical foundation for selecting appropriate antibiotics combined with antibiotic susceptibility testing. Furthermore, understanding the resistance mechanisms of *E. miricola* through genomic analysis could aid in developing new treatment strategies. However, while mNGS offers the potential to accurately identify pathogens and detect resistance genes, its high cost, complex technical requirements, and dependence on specialized equipment and personnel may make it impractical in resource-poor settings. In contrast, MALDI-TOF/MS is a widely used and cost-effective technique in many medical institutions. Although it has limitations in accurately identifying certain species (such as *E. miricola*) compared to mNGS, its accuracy can be improved through the expansion of databases and the integration of additional biochemical tests or advanced spectral analysis algorithms (13). In addition, a case series has shown that Fourier transform infrared spectroscopy (FTIR) is also a promising, cost-effective, and real-time bacterial identification option in resource-limited settings (11).

## 6 Conclusion

Case reports indicate that *E. miricola* infections frequently result in severe complications, including septic shock, as observed in this instance, and are associated with a high mortality rate. The multidrug-resistant nature of the bacteria limits the efficacy of antimicrobial therapy, underscoring the importance of prioritizing its prevention and control. Healthcare professionals should adhere to infection control protocols, including hand hygiene, contact isolation, and thorough environmental cleaning and disinfection. For patients with existing infections, it is imperative to prioritize pathogen examination, particularly mNGS, to facilitate early detection and enable the prompt administration of appropriate antibiotics for targeted treatment, thereby enhancing the likelihood of a favorable prognosis.

## Data Availability

The original contributions presented in the study are included in the article/Supplementary material; further inquiries can be directed to the corresponding author/s.

## References

[1] Li Y Kawamura Y Fujiwara N Naka T Liu H Huang X. *Chryseobacterium miricola* sp. nov., a novel species isolated from condensation water of space station Mir. Syst Appl Microbiol. (2003) 26:523–8. doi: 10.1078/072320203770865828, PMID: 14666980

[2] Green O Murray P Gea-Banacloche JC. Sepsis caused by *Elizabethkingia miricola* successfully treated with tigecycline and levofloxacin. Diagn Microbiol Infect Dis. (2008) 62:430–2. doi: 10.1016/j.diagmicrobio.2008.07.015, PMID: 18842380 PMC2650818

[3] Rahim GR Gupta N. *Elizabethkingia miricola*: discrepancies in identification and antimicrobial susceptibilities. Diagn Microbiol Infect Dis. (2019) 94:104. doi: 10.1016/j.diagmicrobio.2018.11.025, PMID: 30594410

[4] Opota O Diene SM Bertelli C Prod'hom G Eckert P Greub G. Genome of the carbapenemase-producing clinical isolate *Elizabethkingia miricola* EM_CHUV and comparative genomics with Elizabethkingia meningoseptica and *Elizabethkingia anophelis*: evidence for intrinsic multidrug resistance trait of emerging pathogens. Int J Antimicrob Agents. (2017) 49:93–7. doi: 10.1016/j.ijantimicag.2016.09.031, PMID: 27913093

[5] Han MS Kim H Lee Y Kim M Ku NS Choi JY. Relative prevalence and antimicrobial susceptibility of clinical isolates of Elizabethkingia species based on 16S rRNA gene sequencing. J Clin Microbiol. (2016) 55:274–80. doi: 10.1128/JCM.01637-16, PMID: 27847376 PMC5228240

[6] Gupta P Zaman K Mohan B Taneja N. *Elizabethkingia miricola*: a rare non-fermenter causing urinary tract infection. World J Clin Cases. (2017) 5:187–90. doi: 10.12998/wjcc.v5.i5.187, PMID: 28560237 PMC5434319

[7] CLSI. CLSI M100. Performance standards for antimicrobial susceptibility testing. 34th ed. Wayne, PA: Clinical and Laboratory Standards Institute (2024).

[8] Page MJ McKenzie JE Bossuyt PM Boutron I Hoffmann TC Mulrow CD. The PRISMA 2020 statement: an updated guideline for reporting systematic reviews. BMJ. (2021) 372:n71. doi: 10.1136/bmj.n71 33782057 PMC8005924

[9] Munn Z Barker TH Moola S Tufanaru C Stern C McArthur A. Methodological quality of case series studies: an introduction to the JBI critical appraisal tool. JBI Evid Synth. (2020) 18:2127–33. doi: 10.11124/JBISRIR-D-19-00099, PMID: 33038125

[10] Soler-Iborte E Rivera-Izquierdo M Valero-Ubierna C. Opportunistic *Elizabethkingia miricola* infections in intensive care unit. Spain Emerg Infect Dis. (2024) 30:834–7. doi: 10.3201/eid3004.231491, PMID: 38526191 PMC10977848

[11] Rodríguez-Temporal D García-Cañada JE Candela A Oteo-Iglesias J Serrano-Lobo J Pérez-Vázquez M. Characterization of an outbreak caused by *Elizabethkingia miricola* using Fourier-transform infrared (FTIR) spectroscopy. Eur J Clin Microbiol Infect Dis. (2024) 43:797–803. doi: 10.1007/s10096-024-04764-4, PMID: 38356016

[12] Wei Q Zuo W Cong R Luo K Dong S. Combination therapy of trimethoprim-sulfamethoxazole (TMP-SMZ) and Eravacycline for treating *Elizabethkingia anophelis*-induced pulmonary infections: a case report. Infect Drug Resist. (2024) 17:4825–32. doi: 10.2147/IDR.S490902, PMID: 39512396 PMC11542489

[13] Yung CF Maiwald M Loo LH Soong HY Tan CB Lim PK. Elizabethkingia anophelis and association with tap water and handwashing. Singapore Emerg Infect Dis. (2018) 24:1730–3. doi: 10.3201/eid2409.171843, PMID: 30124415 PMC6106401

