# Supplementary material for: Septic shock caused by Elizabethkingia miricola in an elderly trauma patient: a case report and systematic literature review
Source: Front Med (Lausanne). 2025 May 7;12:1561379. doi: 10.3389/fmed.2025.1561379 (PMC12094253; doi:10.3389/fmed.2025.1561379)
Supplement: Supplementary file 1 [file Table_1.DOCX]

**Suppl. Table 1|** Patient characteristics of included studies.

| Year/  Country | Case/  Age/sex | Site of infection | Clinical symptoms | Primary disease | Sensitive drug | Anti-infection protocol | Bacterial eradication | Prognosis | Nosocomial infection |
| --- | --- | --- | --- | --- | --- | --- | --- | --- | --- |
| 2008[1]  The USA | 1/55/M | Blood,lung | sepsis, respiratory insufficiency | Mantle cell lymphoma | ciprofloxacin, levofloxacin | Tigecycline, levofloxacin | NO | Death | Yes |
| 2015[2]  Italy | 1/34/F | Blood | Abdomen hemorrhage, respiratory distress | Alcoholic pancreatitis | levofloxacin, ciprofloxacin, TMP-SMX and piperacillin. | Ciprofloxacin, imipenem | eradication | Favourable | Unknown |
| 2016[3]  Italy | 1/2/M | Urinary tract | Fever and severe clinical conditions | Spina bifida, bladder exstrophy, atelectasis, tracheostomy, chronic kidney insufficiency, Mitrofanoff stoma fistula | Unknown | Unknown | Unknown | Unknown | Unknown |
| 2017[4]  Denmark | 1/64/M | Knee joint | increased pain, warm and sore knee | recurrent erysipelas of both legs | ciprofloxacin and cotrimoxazole | piperacillin-tazobactam | Unknown | Favourable | Yes |
| 2017[5]  Switzerl-and | 1/82/M | lung | Severe nosocomial pneumonia | Recurrent pneumonia | Amikacin, Gentamicin, Minocycline, Levofloxacin | Amikacin, imipenem | NO | Death | Yes |
| 2017[6]  South Korea | 18/  Unknown/Unknown | Lower respiratory（12） Blood（2）Other(4) | Unknown | Unknown | piperacillin-tazobactam (17/18), ciprofloxacin (10/18), levofloxacin (18/18), rifampicin (12/18), gentamicin (8/18) TMP-SMX (5/18) | Unknown | Unknown | Unknown | Unknown |
| 2017[7]  India | 1/25/F | Urinary tract | Difficulty in micturition | bilateral hydroureteronephrosis | gentamicin, ceftriaxone, aztreonam, piperacillin-tazo­bactam and imipenem | piperacillin-tazobactam | eradication | Favourable | No |
| 2017[8]  Polish | 1/35/F | Oral | Periodontitis | Immuno-compromised | ciprofloxacin, levofloxacin, TMP-SMX | levofloxacin | eradication | Favourable | Unknown |
| 2018[9]  The UK | 1/58/M | lung | Pneumothorax，sepsis | Lung transplantation, immunocompromised | piperacillin-tazobactam, amikacin, gentamicin | Tigecycline, piperacillin-tazobactam, amikacin, minocycline | None | Death | Yes |
| 2018[10]  The UK | 1/49/M | lung | Decreased pulmonary function | Cystic fibrosis | piperacillin-tazobactam, ciprofloxacin | ciprofloxacin | eradication | Favourable | No |
| 2020[11]  New Zealand | 1/73/M | Blood | sepsis | hemodialysis | ciprofloxacin, piperacillin-tazobactam | ciprofloxacin | eradication | Favourable | No |
| 2020[12]  France | 1/69/M | Blood | hemodynamic dysfunction | Acute pancreatitis, peritoneal dialysis | piperacillin-tazobactam, ciprofloxacin, levofloxacin, minocycline | piperacillin-tazobactam | eradication | Favourable | Yes |
| 2020[13]  Spain | 1/52/F | Right ankle | Pain,  Slight subsidence | Right foot injury | Ciprofloxacin, levofloxacin, piperacillin-tazobactam | piperacillin-tazobactam, oral levofloxacin | unknown | favourable | Yes |
| 2021[14]  China | 1/59/M | Urinary tract | Unknown | Unknown | Carrying Gene (blaGOB-13, blaB-6, vanW, adeF, cmeB), associated with resistance to β-lactam, vancomycin, and multidrug resistance efflux pumps | Unknown | Unknown | Favourable | Unknown |
| 2021[15]  China | 1/24/M | Intracranial | Headache, vomiting | intracranial infection | Ciprofloxacin, TMP-SMX, Tigecycline,  Cefoperazone/sulbactam, Levofloxacin | Cefoperazone/sulbactam, moxifloxacin | Unknown | Death | No |
| 2022[16]  The USA | 1/67/M | Urinary tract, bone marrow | left thigh pain, erythema, swelling, and tenderness | stage 3 chronic kidney disease, prostate cancer, urethral stricture requiring placement of a supra pubic catheter | Minocycline: sensitivity, Levofloxacin: intermediate sensitivity | Aztreonam, Tigecycline | eradication | Unknown | Unknown |
| 2023[17]  Australia | 1/80s/F | Peritonitis | nausea and lethargy | end-stage kidney disease | ciprofloxacin, piperacillin/tazobactam, gentamicin and TMP-SMX | ciprofloxacin, piperacillin-tazobactam, gentamicin and TMP-SMX | eradication | Favourable | No |
| 2023[18]  China | 1/56/M | Intracranial | Fever and disturbance of consciousness | nasopharyngeal carcinoma | Unknown | cefoperazone/sulbactam | eradication | Favourable | No |
| 2024[19]  China | 1/54/M | Lung | Unknown | Right basal ganglia hemorrhage | Quinolone antibiotics | moxifloxacin | unknown | Death | Yes |
| 2024[20]  Spain | 10/32-74/M (8), F (2) | lung | Tracheobronchitis（6）  Ventilator-associat-ed pneumonia（3）  Bacteremia（1） | COVID-19(8)  Epileptic seizures（1）  Septic shock （1） | Piperacillin/tazobactam: resistance (8)  Carbapenems: resistance (4)  Amikacin: resistance (4) | Unknown | Unknown | Death（5） | Yes |
| 2024[21]  Spain | 17/44-79/M (13), F (4) | Lung (16), blood (1) | Unknown | Unknown | Ciprofoxacin, Levofoxacin, Tigecycline, TMP-SMX | TMP-SMX (12), Levofoxacin(2), piperacillin-tazobactam(2), Ciprofoxacin(1) | Unknown | Favourable(16), not related death(1) | Unknown |

*TMP-SMX: trimethoprim-sulfamethoxazole*

References

1. Green O, Murray P, Gea-Banacloche JC. Sepsis caused by *Elizabethkingia miricola* successfully treated with tigecycline and levofloxacin. *Diagn Microbiol Infect Dis*. (2008) 62:430-432. doi: 10.1016/j.diagmicrobio.2008.07.015
2. Rossati A, Kroumova V, Bargiacchi O, Brustia D, Luigi Garavelli P. *Elizabethkingia miricola* bacteriemia in a young woman with acute alcoholic pancreatitis. *Presse Med*. (2015) 44:1071-1072. doi:10.1016/ j.lpm.2015.08.003
3. Colapietro M, Endimiani A, Sabatini A, Marcoccia F, Celenza G, Segatore B, et al. BlaB-15, a new BlaB metallo-β-lactamase variant found in an *Elizabethkingia miricola* clinical isolate. *Diagn Microbiol Infect Dis*. (2016) 85:195-197. doi:10.1016/ j. diagmicrobio.2015.11.016
4. Eriksen HB, Gumpert H, Faurholt CH, Westh H. Determination of Elizabethkingia Diversity by MALDI-TOF Mass Spectrometry and Whole-Genome Sequencing. *Emerg Infect Dis.* (2017) 23:320-323. doi:10.3201/eid2302.161321
5. Opota O, Diene SM, Bertelli C, Prod'hom G, Eckert P, Greub G. Genome of the carbapenemase-producing clinical isolate *Elizabethkingia miricola* EM_CHUV and comparative genomics with *Elizabethkingia meningoseptica* and *Elizabethkingia anophelis*: evidence for intrinsic multidrug resistance trait of emerging pathogens. *Int J Antimicrob Agents*. (2017) 49:93-97. doi:10.1016/ j. ijantimicag.2016.09.031
6. Han MS, Kim H, Lee Y, Kim M, Ku NS, Choi JY, et al. Relative Prevalence and Antimicrobial Susceptibility of Clinical Isolates of Elizabethkingia Species Based on 16S rRNA Gene Sequencing. *J Clin Microbiol*. (2016) 55:274-280. doi:10.1128/JCM.01637-16
7. Gupta P, Zaman K, Mohan B, Taneja N. *Elizabethkingia miricola*: A rare non-fermenter causing urinary tract infection. *World J Clin Cases.* (2017) 5:187-190. doi:10.12998/wjcc. v5.i5.187
8. Zdziarski P, Paściak M, Rogala K, Korzeniowska-Kowal A, Gamian A. *Elizabethkingia miricola* as an opportunistic oral pathogen associated with superinfectious complications in humoral immunodeficiency: a case report. *BMC Infect Dis.* (2017) 17:763. doi:10.1186/s12879-017-2886-7
9. Monteagudo Vela M, Zych B, García Saez D, Simon AR. Fatal infection with *Elisabethkingia miricola* after lung transplantation. *J Hosp Infect.* (2018) 100: e259-e260. doi: 10.1016/j.jhin.2018.07.015
10. Frost F, Nazareth D. Case Report: First report of *Elizabethkingia miricola* infection in a patient with cystic fibrosis. *F1000Res.* (2018) 7:440. doi:10.12688/f1000research.14441.2
11. Howard JC, Chen K, Anderson T, Dalton SC. *Elizabethkingia miricola* bacteraemia in a haemodialysis patient. *Access Microbiol.* (2020) 2: acmi000098. doi:10.1099/acmi.0.000098
12. Penven M, Lalieu A, Boruchowicz A, Paluch M, Diedrich T, Dewulf G, et al. Bacteremia caused by *Elizabethkingia miricola* in a patient with acute pancreatitis and peritoneal dialysis. *Med Mal Infect.* (2020) 50:379-381. doi: 10.1016/j.medmal.2020.01.009
13. Calatrava E, Casanovas I, Foronda C, Cobo F. Joint infection due to *Elizabethkingia miricola.* *Rev Esp Quimioter.* (2020) 33:141-142. doi:10.37201/req/081.2019
14. Yang C, Liu Z, Yu S, Ye K, Li X, Shen D. Comparison of three species of Elizabethkingia genus by whole-genome sequence analysis. *FEMS Microbiol Lett.* (2021) 368: fnab018. doi:10.1093/femsle/fnab018
15. Gao H, Li T, Feng L, Zhang S. *Elizabethkingia miricola* Causes Intracranial Infection: A Case Study. *Front Med (Lausanne).* (2021) 8:761924. doi:10.3389/fmed.2021.761924
16. Badawi K, Deskins S, Catherman K, Lastinger A. Out of this world: *Elizabethkingia miricola* complicated urinary tract infection in a patient with associated pubic symphysis osteomyelitis and pyomyositis. *IDCases*. (2022) 29: e01573. doi: 10.1016/j.idcr.2022.e01573
17. Kayes M, Potter D, Wong J, Spicer T. Peritoneal dialysis-associated peritonitis with *Elizabethkingia miricola*. *BMJ Case Rep.* (2023) 16: e255491. doi:10.1136/bcr-2023-255491
18. Zhuo X, Zhou Y, Liu L. Acute bacterial encephalitis complicated with recurrent nasopharyngeal carcinoma associated with *Elizabethkingia miricola* infection: A case report. *Front Neurol.* (2023) 13:965939. doi:10.3389/fneur.2022.965939
19. Qi PQ, Zeng YJ, Peng W, Kuai J. Lung imaging characteristics in a patient infected with *Elizabethkingia miricola* following cerebral hemorrhage surgery: A case report. *World J Clin Cases.* (2024) 12:169-175. doi:10.12998/wjcc. v12.i1.169
20. Soler-Iborte E, Rivera-Izquierdo M, Valero-Ubierna C. Opportunistic *Elizabethkingia miricola* Infections in Intensive Care Unit, Spain. *Emerg Infect Dis.* (2024) 30:834-837. doi:10.3201/eid3004.231491
21. Rodríguez-Temporal D, García-Cañada JE, Candela A, Oteo-Iglesias J, Serrano-Lobo J, Pérez-Vázquez M, et al. Characterization of an outbreak caused by *Elizabethkingia miricola* using Fourier-transform infrared (FTIR) spectroscopy. *Eur J Clin Microbiol Infect Dis*. (2024) 43:797-803. doi:10.1007/s10096-024-04764-4
