# Supplementary material for: Septic shock caused by Elizabethkingia miricola in an elderly trauma patient: a case report and systematic literature review
Source: Front Med (Lausanne). 2025 May 7;12:1561379. doi: 10.3389/fmed.2025.1561379 (PMC12094253; doi:10.3389/fmed.2025.1561379)
Supplement: Supplementary file 2 [file Table_2.DOCX]

**Suppl. Table 2|** Quality assessment of single-case reports.

| Author | Year | Were patient’s demographic characteristics clearly described? | Was the patient’s history clearly described and presented as a timeline? | Was the current clinical condition of the patient on presentation clearly described? | Were diagnostic tests or assessment methods and the results clearly described? | Was the intervention(s) or treatment procedure(s) clearly described? | Was the post-intervention clinical condition clearly described? | Were adverse events(harms) or unanticipated events identified and described? | Does the case report provide takeaway lessons? | Risk of bias |
| --- | --- | --- | --- | --- | --- | --- | --- | --- | --- | --- |
| Green, O., et al. | 2008[1] | Yes | Yes | Yes | Yes | Yes | Yes | No | Yes | Low |
| Rossati, A., et al. | 2015[2] | Yes | No | Yes | Yes | Yes | Yes | No | Yes | Low |
| Colapietro, M., et al. | 2016[3] | Yes | No | Yes | Yes | No | No | No | Yes | High |
| Eriksen, H.B., et al. | 2017[4] | Yes | Yes | Yes | Yes | Yes | Yes | No | Yes | Low |
| Opota, O., et al. | 2017[5] | Yes | No | Yes | Yes | Yes | Yes | No | Yes | Low |
| Gupta, P., et al. | 2017[6] | Yes | No | Yes | Yes | Yes | Yes | No | Yes | Low |
| Zdziarski, P., et al. | 2017[7] | Yes | Yes | Yes | Yes | Yes | Yes | No | Yes | Low |
| Monteagudo Vela, M., et al. | 2018[8] | Yes | Yes | Yes | Yes | Yes | Yes | No | Yes | Low |
| Frost, F., et al. | 2018[9] | Yes | Yes | Yes | Yes | Yes | Yes | No | Yes | Low |
| Howard, J.C., et al. | 2020[10] | Yes | Yes | Yes | Yes | Yes | Yes | No | Yes | Low |
| Penven, M., et al. | 2020[11] | Yes | Yes | Yes | Yes | Yes | Yes | No | Yes | Low |
| Calatrava, E., et al. | 2020[12] | Yes | Yes | No | Yes | Yes | Yes | Yes | Yes | Low |
| Yang, C., et al. | 2021[13] | Yes | No | No | No | No | No | No | Yes | High |
| Gao, H., et al. | 2021[14] | Yes | Yes | Yes | Yes | Yes | Yes | No | Yes | Low |
| Badawi, K., et al. | 2022[15] | Yes | Yes | Yes | Yes | Yes | No | No | Yes | Low |
| Kayes, M., et al. | 2023[16] | No | Yes | Yes | Yes | Yes | Yes | No | Yes | Low |
| Zhuo, X., et al. | 2023[17] | Yes | Yes | Yes | Yes | Yes | Yes | No | Yes | Low |
| Qi, P.Q., et al. | 2024[18] | Yes | Yes | Yes | Yes | Yes | Yes | No | Yes | Low |

References

1. Green O, Murray P, Gea-Banacloche JC. Sepsis caused by *Elizabethkingia miricola* successfully treated with tigecycline and levofloxacin. *Diagn Microbiol Infect Dis*. (2008) 62:430-432. doi: 10.1016/j.diagmicrobio.2008.07.015
2. Rossati A, Kroumova V, Bargiacchi O, Brustia D, Luigi Garavelli P. *Elizabethkingia miricola* bacteriemia in a young woman with acute alcoholic pancreatitis. *Presse Med*. (2015) 44:1071-1072. doi:10.1016/ j.lpm.2015.08.003
3. Colapietro M, Endimiani A, Sabatini A, Marcoccia F, Celenza G, Segatore B, et al. BlaB-15, a new BlaB metallo-β-lactamase variant found in an *Elizabethkingia miricola* clinical isolate. *Diagn Microbiol Infect Dis*. (2016) 85:195-197. doi:10.1016/ j. diagmicrobio.2015.11.016
4. Eriksen HB, Gumpert H, Faurholt CH, Westh H. Determination of Elizabethkingia Diversity by MALDI-TOF Mass Spectrometry and Whole-Genome Sequencing. *Emerg Infect Dis.* (2017) 23:320-323. doi:10.3201/eid2302.161321
5. Opota O, Diene SM, Bertelli C, Prod'hom G, Eckert P, Greub G. Genome of the carbapenemase-producing clinical isolate *Elizabethkingia miricola* EM_CHUV and comparative genomics with *Elizabethkingia meningoseptica* and *Elizabethkingia anophelis*: evidence for intrinsic multidrug resistance trait of emerging pathogens. *Int J Antimicrob Agents*. (2017) 49:93-97. doi:10.1016/ j. ijantimicag.2016.09.031
6. Gupta P, Zaman K, Mohan B, Taneja N. *Elizabethkingia miricola*: A rare non-fermenter causing urinary tract infection. *World J Clin Cases.* (2017) 5:187-190. doi:10.12998/wjcc. v5.i5.187
7. Zdziarski P, Paściak M, Rogala K, Korzeniowska-Kowal A, Gamian A. *Elizabethkingia miricola* as an opportunistic oral pathogen associated with superinfectious complications in humoral immunodeficiency: a case report. *BMC Infect Dis.* (2017) 17:763. doi:10.1186/s12879-017-2886-7
8. Monteagudo Vela M, Zych B, García Saez D, Simon AR. Fatal infection with *Elisabethkingia miricola* after lung transplantation. *J Hosp Infect.* (2018) 100: e259-e260. doi: 10.1016/j.jhin.2018.07.015
9. Frost F, Nazareth D. Case Report: First report of *Elizabethkingia miricola* infection in a patient with cystic fibrosis. *F1000Res.* (2018) 7:440. doi:10.12688/f1000research.14441.2
10. Howard JC, Chen K, Anderson T, Dalton SC. *Elizabethkingia miricola* bacteraemia in a haemodialysis patient. *Access Microbiol.* (2020) 2: acmi000098. doi:10.1099/acmi.0.000098
11. Penven M, Lalieu A, Boruchowicz A, Paluch M, Diedrich T, Dewulf G, et al. Bacteremia caused by *Elizabethkingia miricola* in a patient with acute pancreatitis and peritoneal dialysis. *Med Mal Infect.* (2020) 50:379-381. doi: 10.1016/j.medmal.2020.01.009
12. Calatrava E, Casanovas I, Foronda C, Cobo F. Joint infection due to *Elizabethkingia miricola.* *Rev Esp Quimioter.* (2020) 33:141-142. doi:10.37201/req/081.2019
13. Yang C, Liu Z, Yu S, Ye K, Li X, Shen D. Comparison of three species of Elizabethkingia genus by whole-genome sequence analysis. *FEMS Microbiol Lett.* (2021) 368: fnab018. doi:10.1093/femsle/fnab018
14. Gao H, Li T, Feng L, Zhang S. *Elizabethkingia miricola* Causes Intracranial Infection: A Case Study. *Front Med (Lausanne).* (2021) 8:761924. doi:10.3389/fmed.2021.761924
15. Badawi K, Deskins S, Catherman K, Lastinger A. Out of this world: *Elizabethkingia miricola* complicated urinary tract infection in a patient with associated pubic symphysis osteomyelitis and pyomyositis. *IDCases*. (2022) 29: e01573. doi: 10.1016/j.idcr.2022.e01573
16. Kayes M, Potter D, Wong J, Spicer T. Peritoneal dialysis-associated peritonitis with *Elizabethkingia miricola*. *BMJ Case Rep.* (2023) 16: e255491. doi:10.1136/bcr-2023-255491
17. Zhuo X, Zhou Y, Liu L. Acute bacterial encephalitis complicated with recurrent nasopharyngeal carcinoma associated with *Elizabethkingia miricola* infection: A case report. *Front Neurol.* (2023) 13:965939. doi:10.3389/fneur.2022.965939
18. Qi PQ, Zeng YJ, Peng W, Kuai J. Lung imaging characteristics in a patient infected with *Elizabethkingia miricola* following cerebral hemorrhage surgery: A case report. *World J Clin Cases.* (2024) 12:169-175. doi:10.12998/wjcc. v12.i1.169
