# Supplementary material for: Septic shock caused by Elizabethkingia miricola in an elderly trauma patient: a case report and systematic literature review
Source: Front Med (Lausanne). 2025 May 7;12:1561379. doi: 10.3389/fmed.2025.1561379 (PMC12094253; doi:10.3389/fmed.2025.1561379)
Supplement: Supplementary file 3 [file Table_3.DOCX]

**Suppl. Table 3|** Quality assessment of case series.

| Author/Year | Han M-S., et al./2017[1] | Soler-Iborte, E., et al./2024[2] | Rodríguez-Temporal, D., et al./2024[3] |
| --- | --- | --- | --- |
| 1. Were there clear criteria for inclusion in the case series? | No | Yes | No |
| 2. Was the condition measured in a standard, reliable way for all participants included in the case series? | Yes | Yes | Yes |
| 3. Were valid methods used for identification of the condition for all participants included in the case series? | Yes | Yes | Yes |
| 4. Did the case series have consecutive inclusion of participants? | Yes | Yes | Yes |
| 5. Did the case series have complete inclusion of participants? | Yes | Yes | Yes |
| 6. Was there clear reporting of the demographics of the participants in the study? | No | Yes | Yes |
| 7. Was there clear reporting of clinical information of the participants? | No | Yes | No |
| 8. Were the outcomes or follow-up results of cases clearly reported? | No | Yes | Yes |
| 9. Was there clear reporting of the presenting sites’/clinics’ demographic information? | Yes | Yes | Yes |
| 10. Was statistical analysis appropriate? | Yes | Yes | Yes |
| Risk of Bias | High | Low | Low |

References

1. Han MS, Kim H, Lee Y, Kim M, Ku NS, Choi JY, et al. Relative Prevalence and Antimicrobial Susceptibility of Clinical Isolates of Elizabethkingia Species Based on 16S rRNA Gene Sequencing. *J Clin Microbiol*. (2016) 55:274-280. doi:10.1128/JCM.01637-16
2. Soler-Iborte E, Rivera-Izquierdo M, Valero-Ubierna C. Opportunistic *Elizabethkingia miricola* Infections in Intensive Care Unit, Spain. *Emerg Infect Dis.* (2024) 30:834-837. doi:10.3201/eid3004.231491
3. Rodríguez-Temporal D, García-Cañada JE, Candela A, Oteo-Iglesias J, Serrano-Lobo J, Pérez-Vázquez M, et al. Characterization of an outbreak caused by *Elizabethkingia miricola* using Fourier-transform infrared (FTIR) spectroscopy. *Eur J Clin Microbiol Infect Dis*. (2024) 43:797-803. doi:10.1007/s10096-024-04764-4
